# European Code Against Cancer, 5th edition – ultraviolet radiation, radon and cancer

**DOI:** 10.1002/1878-0261.70171

**Published:** 2026-01-16

**Authors:** David Ritchie, Quentin Crowley, Rüdiger Greinert, Maria Albin, Isabelle Baldi, Dario Consonni, Béatrice Fervers, Gerard Hoek, Sylvia H. J. Jochems, Martin Röösli, Martie van Tongeren, Nadia Vilahur, Ariadna Feliu, Hajo Zeeb, Joachim Schüz, Erica D'Souza, Carolina Espina, Hans Kromhout

**Affiliations:** ^1^ Environmental and Lifestyle Epidemiology Branch International Agency for Research on Cancer Lyon France; ^2^ Geology, School of Natural Sciences Trinity College Dublin Ireland; ^3^ Trinity Centre for the Environment Trinity College Dublin Ireland; ^4^ Department of Molecular Cell Biology, Skin Cancer Center Elbe Kliniken Buxtehude Germany; ^5^ Institute for Environmental Medicine Karolinska Institutet Stockholm Sweden; ^6^ Department of Laboratory Medicine Lund University Sweden; ^7^ Univ. Bordeaux, Inserm U1219, Bordeaux Population Health Research Centre, EPICENE Team Bordeaux France; ^8^ Service Santé Travail Environment, Pôle de Santé Publique, CHU de Bordeaux France; ^9^ Occupational Health Unit, Fondazione IRCCS Ca' Granda Ospedale Maggiore Policlinico Milan Italy; ^10^ Department of Prevention Cancer Environment, Centre Léon Bérard Lyon France; ^11^ Inserm U1296, “Radiation: Defense, Health and Environment” Lyon France; ^12^ Institute for Risk Assessment Sciences Utrecht University The Netherlands; ^13^ Swiss Tropical and Public Health Institute (Swiss TPH) Allschwil Switzerland; ^14^ University of Basel Switzerland; ^15^ Centre for Occupational and Environmental Health, School of Health Sciences University of Manchester UK; ^16^ European Agency for Safety and Health at Work (EU‐OSHA) Bilbao Spain; ^17^ Department of Primary Care and Public Health, School of Public Health Imperial College London UK; ^18^ Department of Prevention and Evaluation Leibniz – Institute for Prevention Research and Epidemiology – BIPS GmbH Bremen Germany; ^19^ Health Sciences Bremen University of Bremen Germany

**Keywords:** cancer prevention, cutaneous melanoma, European Code Against Cancer, indoor tanning devices, lung cancer, public health policy, radon, skin cancer, ultraviolet radiation

## Abstract

The European Code Against Cancer (ECAC) provides evidence‐based recommendations to help individuals reduce their cancer risk. For the 5th edition (ECAC5), recommendations on ultraviolet radiation (UVR) and indoor radon exposures were updated, and complementary recommendations for policymakers were introduced. UVR and radon are classified as carcinogenic to humans (group 1 carcinogens) in the International Agency for Research on Cancer (IARC) Monographs. Solar UVR and, to a lesser extent, artificial forms of UVR exposure are major causes of skin cancer, while radon gas is a leading cause of lung cancer. This paper summarises the evidence for retaining and refining these recommendations. For individuals, ECAC5 advises avoiding excessive sun exposure, especially in children, using sun protection, and never using sunbeds; for radon, checking local radon maps, seeking professional measurement where appropriate and taking remedial action, if necessary, are recommended. For policymakers, ECAC5 encourages harmonised UVR protection measures across the European Union, enforcement of regulations concerning indoor tanning devices, and enabling access to testing of radon levels, and support for mitigation and remediation. These recommendations provide actionable, evidence‐based recommendations to help reduce cancer risk and align with Europe's Beating Cancer Plan.

## Graphical abstract

Here we update the cancer prevention recommendations on ultraviolet radiation and indoor radon gas, listed at positions 8 and 10 of the 5th edition of the European Code Against Cancer (ECAC5). Individuals are advised to avoid too much sun, use sun protection and never use sunbeds. They are also advised to check local radon maps and fix high levels at home, if needed. Policymakers are encouraged to tighten sunbed controls and strengthen EU‐wide protections against ultraviolet radiation and radon.

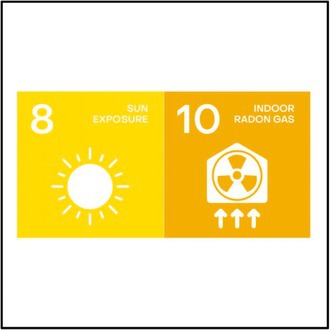

**Abbreviations**
aERR: adjusted Excess Relative Risk
APC: annual percentage change
ASR: Age‐Standardised Rate
BCC: basal cell carcinoma
Bq·m^−3^
: becquerels per cubic metre
BSS: basic safety standard directive
CI: confidence interval
CM: cutaneous melanoma
DNA: deoxyribonucleic acid
EBCP: Europe's Beating Cancer Plan
ECAC: European Code Against Cancer
ECAC4: European Code Against Cancer, 4th edition
ECAC5: European Code Against Cancer, 5th edition
EEA: European Environment Agency
EOR: excess odds ratio
EU: European Union
EU‐OSHA: European Agency for Safety and Health at Work
GBD: global burden of disease
IAEA: International Atomic Energy Agency
IARC Monographs: IARC monographs on the identification of carcinogenic hazards to humans
IARC: International Agency for Research on Cancer
ICRP: International Commission on Radiological Protection
JRC: Joint Research Centre
LNT: linear‐no‐threshold
nm: nanometres
NMSC: non‐melanoma skin cancer
OR: odds ratio
OSH: occupational safety and health
PAF: population attributable fraction
RR: relative risk
SCC: squamous cell carcinoma
SCHEER: Scientific Committee on Health, Environmental and Emerging Risks
SIR: standardised incidence ratio
SPF: sun protection factor
TECDOC: technical document
UV: ultraviolet
UVA: ultraviolet A
UVB: ultraviolet B
UVI: Global Solar UV Index
UVR: ultraviolet radiation
WHO: World Health Organization

## 1 Introduction

The European Code Against Cancer (ECAC) is an initiative of the European Commission that provides evidence‐based cancer prevention recommendations for the public [1]. The latest 5th edition (ECAC5) has been coordinated by the International Agency for Research on Cancer (IARC) as part of the World Code Against Cancer Framework [2], under which region‐specific codes are developed following a standardised methodology as described in Espina et al. [3].

**Fig. 1:**
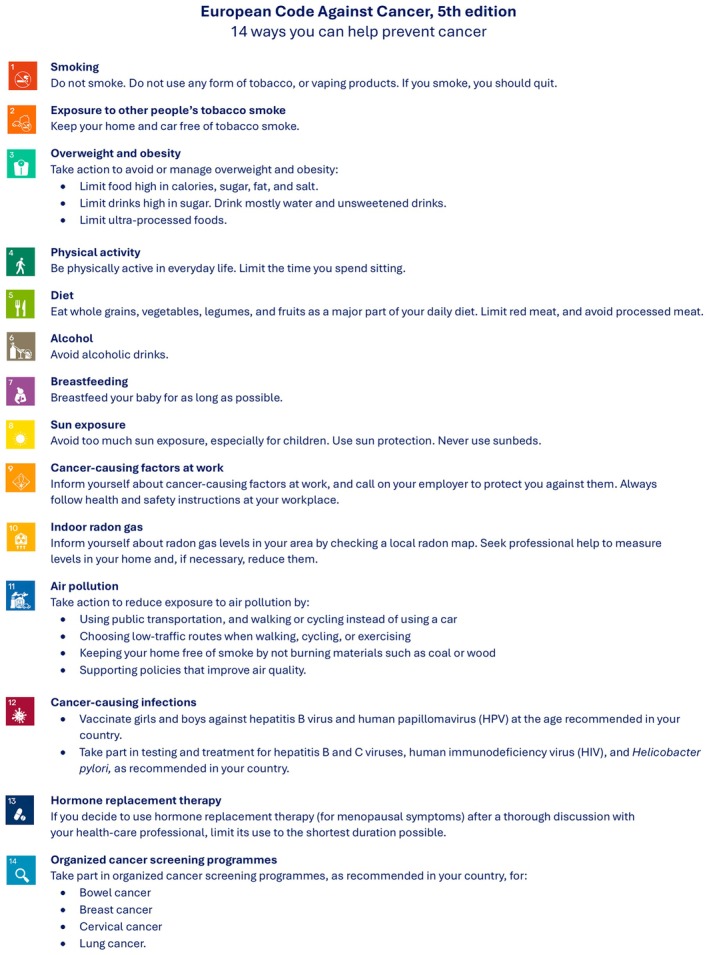
European Code Against Cancer, 5th edition: recommendations for individuals. The 14 recommendations of the European Code Against Cancer, 5th edition (ECAC5) adopted by the Scientific Committee of the ECAC5 project. © 2026 International Agency for Research on Cancer / WHO. Used with permission.

ECAC5 distils the latest scientific evidence arising since the 2014 publication of the ECAC's 4th edition (ECAC4) to propose 14 cancer prevention recommendations (Fig. 1). ECAC5 differs from previous editions by also targeting European Union (EU) policymakers with 14 complementary population‐level recommendations [4]. The full text of ECAC5 is presented in Annex S1. This article presents the rationale and justification for updating the ECAC4 recommendations concerning exposure to ultraviolet radiation (UVR) and indoor radon.

Other types of ionising radiation, although also established to be carcinogenic [5], were not considered for ECAC5 due to low public exposure or the lack of feasible opportunities to prevent exposure. Non‐ionising electromagnetic fields were not included given the limited evidence of carcinogenicity in humans [6, 7, 8]. Occupational exposure to ionising radiation is formally addressed in a separate recommendation described in Jochems et al. [9].

## 2 Ultraviolet radiation and radon exposure in the European Union/Europe

### 2.1 Ultraviolet radiation

UVR is a part of the electromagnetic spectrum. Sunlight is the principal source of UVR in daily life, although artificial sources for medical, industrial or cosmetic purposes provide further exposure for certain population groups. Solar UVR and UV‐emitting indoor tanning devices have both been classified as ‘carcinogenic to humans’ (Group 1) by the IARC Monographs on the Identification of Carcinogenic Hazards to Humans (IARC Monographs) [5]. The established biological properties of UVR are described in Greinert et al., which details the scientific justification for the ECAC4 recommendation on UVR [10].

The irradiance of solar UVR is influenced by various factors including time of day, season, latitude, altitude, cloud cover and air pollution. Its intensity at the Earth's surface can be measured using the Global Solar UV Index (UVI), which provides a standardised measure of solar UVR [11]: the higher the index, the greater and more rapid the potential harm to skin and eyes. Sun protection measures are recommended once the UVI reaches 3 or higher [10]. As an illustrative example, the global UVI forecast at 12:00 UTC on 13 June 2025 is provided in Fig. S1.

UVR increased in southern and central Europe since the 1990s [12]. Recent monitoring data from central Europe indicates increases in UV exposure in the range of ~10–20% since the 1990s [13].

In eastern Europe, decreased ozone and cloud cover led to daily UV radiation increasing by 5–8% per decade [14]. Long‐term atmospheric monitoring data have indicated that UVI has increased across western Europe, but such trends are not replicated in high‐latitude northern European settings [15, 16]. Nevertheless, high values of the UVI have been periodically recorded across Nordic countries, for example during the summer of 2018. This phenomenon has been linked to regular clear skies, dry conditions and heatwaves, which have become more common in recent years [17].

Occupational exposure to solar and artificial UVR is an important concern. Occupational exposure to UVR is mostly attributable to outdoor work, during which individuals may be exposed without adequate protection to solar UVR for extended periods of time. According to data from a workers' survey conducted by the European Agency for Safety and Health at Work (EU‐OSHA) in 2023 across six EU countries, 20% of workers face substantial occupational exposure to solar UVR [18]. Artificial sources of UVR in an occupational setting include various forms of lamps used in surface‐coating, medical, cosmetic and food‐hygiene applications which expose between 1.5% and 3.3% of workers in the EU [19]. Further details of occupational exposure to carcinogens and the associated ECAC5 recommendation are addressed in Jochems et al. [9].

UV‐emitting indoor tanning devices deliver UVR equivalent to a UVI of 12, equivalent to midday sun at the Equator. While the UVR may vary across the devices, there is an increasing tendency towards higher UV irradiance [20]. Usage also varies by country. A 2014 meta‐analysis covering 16 countries globally (including Denmark, France, Germany, Ireland, Sweden and the United Kingdom) reported that 35.7% of adults and 19.3% of adolescents in the included studies had used indoor tanning devices at least once [21]. A more recent 2019 study of 227 888 individuals participating in a skin cancer campaign in 30 European countries found that the prevalence of ever use of indoor tanning devices was considerably lower at 10.6%. The prevalence of use at a country level ranged from 0.5% (95% CI, 0.1–1.7) in Malta to 26.5% (95% CI, 25.6–27.4) in Belgium [22]. Data from Germany show an overall decline in the current use of indoor tanning devices for people aged between 14 and 45 years from 14.6% (95% CI, 13.6–15.6) in 2012 to 6.5% (95% CI, 5.4–7.4) in 2022 [23], which in part may be due to the COVID‐19 pandemic.

### 2.2 Radon

Radon‐222 is a naturally occurring radioactive gas that occurs as an intermediate decay product in the uranium‐238 decay chain and is immediately preceded by radium‐226 [24]. It can infiltrate buildings through structural gaps in the basement and accumulate in confined and poorly ventilated spaces [25]. As a consequence, thermal insulation measures without considering appropriate radon protection have been observed to lead to increased indoor radon levels [26]. Indoor concentration depends on a variety of factors including geology, sub‐surface gaseous permeability, ventilation and building materials [27, 28]. Although residential settings remain the primary source of radon exposure for most people, occupational exposure to radon can occur in any workplace, particularly those located in high radon areas, or in buildings or rooms with poor ventilation [29, 30, 31]. Mining and water treatment sectors are deemed at elevated occupational radon exposure risk [32, 33], but any workplace in a basement setting, such as libraries, schools, universities and cultural centres, may also pose an elevated occupational radon exposure risk [29, 34].

**Fig. 2:**
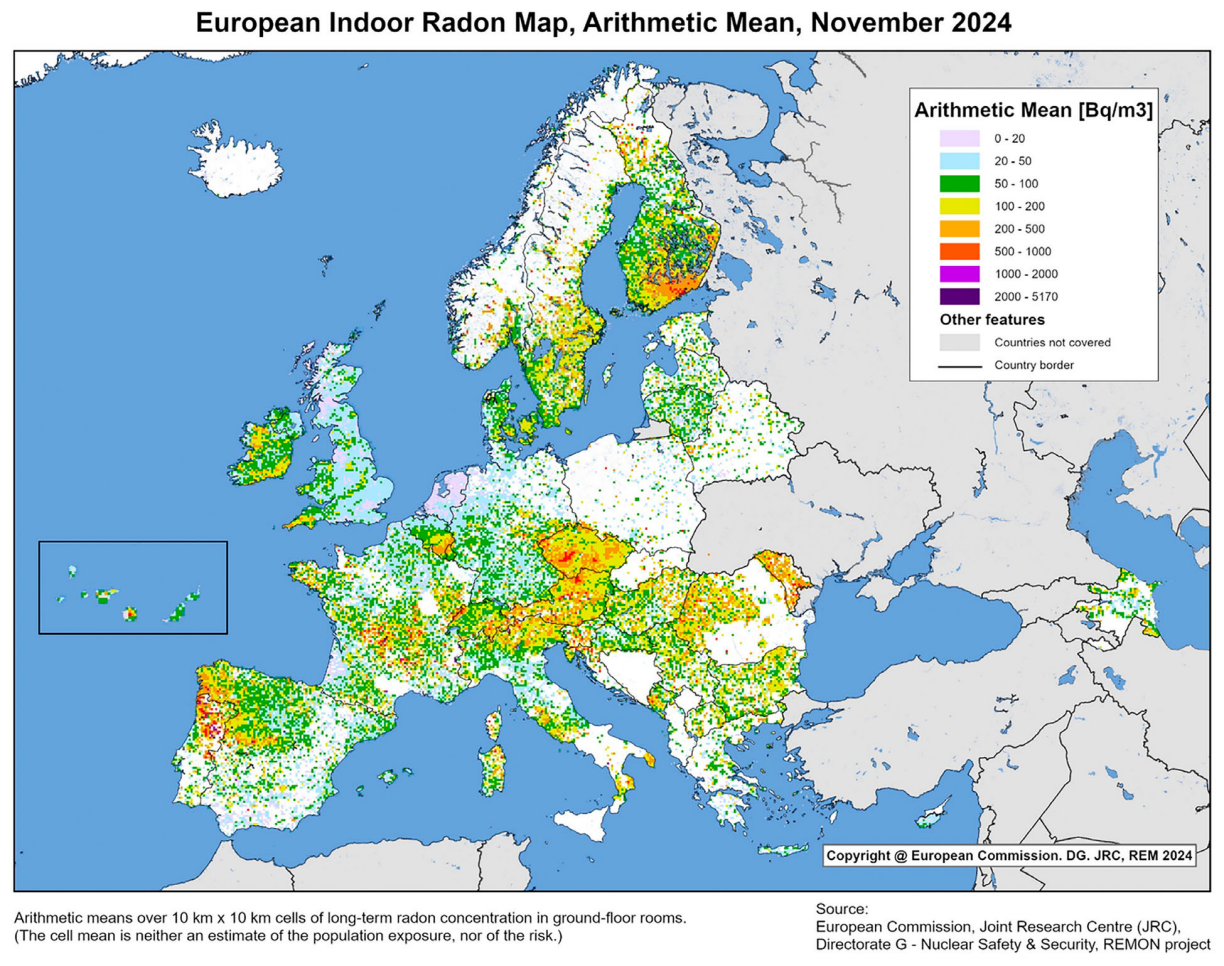
European indoor radon concentration map (November 2024). Concentration is measured in ground‐floor rooms and aggregated into uniform 10 km × 10 km grids according to available data supplied by national authorities. The colour of each cell corresponds to its average radon concentration. © European Union, 2024. Reuse authorised under the Creative Commons Attribution 4.0 International (CC BY 4.0) licence.

Radon levels vary across Europe with concentrations highest in areas with uranium‐rich bedrock, Quaternary deposits or soil, including but not limited to areas in the Czech Republic, Finland, Germany, Ireland, Portugal and Switzerland [32, 35, 36, 37]. Indoor radon concentration is measured in becquerels per cubic metre (Bq·m^−3^). The WHO recommends that indoor radon levels do not exceed 100 Bq·m^−3^ [38]. As typical indoor radon concentration in dwellings varies, EU legislation has been enacted to set a recommended upper limit for Member States of 300 Bq·m^−3^ [39]. The Joint Research Centre (JRC) of the European Commission has developed a programme to periodically map indoor radon exposure at the European level. Figure 2 presents the most recent (at the time of writing) annual indoor radon concentrations in Europe (2024) [40].

## 3 Cancer burden in the European Union/Europe attributable to ultraviolet radiation and radon exposure

### 3.1 Ultraviolet radiation

The European Environment Agency (EEA) estimated that between 3% and 4% of all cancer cases in Europe may be attributable to UVR [41]. Much of this is attributed to skin cancer for which UVR is the major cause. UVR has the potential to both initiate skin cancer by causing DNA damage leading to mutations causing cancer and it can also promote skin cancer development by suppressing the immune response [42, 43]. For this reason, it is known as a complete carcinogen.

UVR causes cutaneous melanoma (CM), which is the most lethal form of skin cancer. In the EU, there were approximately 101 500 cases (Age‐Standardised Rate [ASR] 11.9/100 000) and 16 700 deaths attributed to CM in 2022 [44]. The number of cases has continued to rise in recent decades. Data from 18 cancer registries in Europe showed that the incidence rate of invasive CM increased annually by 4% in men and 3% in women between 1995 and 2012 [45, 46]. Trends for CM mortality have differed from incidence. Using global burden of disease estimates for 28 European countries (for the years 1992–2021), age‐standardised mortality increased until 2015 (annual percentage change [APC] 0.91; 95% CI, 0.71–1.10) and thereafter declined between 2015 and 2021 (APC −1.82; 95% CI, −3.02 to −0.60) [47]. The divergence reflects the impact of improved early detection, together with advances in treatment that have improved survival.

UVR likewise causes keratinocyte skin cancers, namely, cutaneous squamous‐cell carcinoma (SCC) and basal cell carcinoma (BCC), which are less fatal but occur more frequently than CM [48, 49]. In 2022, an estimated 248 900 cases of NMSC (ASR 16.1/100 000) and 8600 deaths were attributed to NMSC in the EU [44]. However, this may be an underestimate as most cancer registries do not routinely collect or process NMSC data, especially BCC. Country‐level data show an increasing trend of NMSC over recent decades [50, 51]. Between 2013 and 2015 in England, the age‐standardised incidence rates of SCC were 77.3 per 100 000 person‐years in men and 34.1 per 100 000 in women [52]. Projections from the United Kingdom suggest the age‐standardised incidence rate of NMSC will increase by 14% between the years 2023–2025 and 2038‐2040 [53].

In addition to skin cancer, UVR can also cause cancer of the lip, whereas UVR from indoor tanning devices has been linked to ocular melanoma, as well as CM and SCC [5, 20, 54]. A meta‐analysis of epidemiological studies reported an increased risk of CM associated with indoor tanning devices (summary relative risk of 1.20; 95% CI, 1.08–1.34) for ever use compared to never use. First exposure before the age of 35 years was associated with a higher risk (summary relative risk of 1.59; 95% CI, 1.36–1.85) [54]. To place this in context, approximately 3438 CM cases annually across 18 European countries have been estimated to be attributable to use of indoor tanning devices, corresponding to an attributable fraction of 5.4% [54].

### 3.2 Radon

Radon is a key risk factor for lung cancer and is recognised as one of the leading cause of lung cancer among never smokers. Overall, radon is considered the second‐leading cause of lung cancer, following tobacco smoking [38]. The World Health Organization (WHO) estimates that between 3% and 14% of lung cancer cases may be attributed to radon exposure. The proportion attributable to radon exposure is influenced by factors such as the mean concentration of indoor radon, differences in building structures and prevalence of tobacco smoking, which acts synergistically with radon exposure [38]. It has been estimated that around 19 000 lung cancers in Europe were attributable to indoor radon exposure in 2019 [55].

National level estimates indicate that the proportion of lung cancer cases attributable to radon exposure varies across Europe. For example, in Germany, about 2800 lung cancer deaths per year (6.3% of the total) are linked to indoor residential radon exposure [56]. In France, this figure is around 2924 deaths annually, accounting for 9.6% of lung cancer deaths [57]. In Finland, indoor radon is estimated to contribute to 3–8% of lung cancer cases [58], and in Ireland, studies suggest that approximately 13% of lung cancer cases, or about 350 cases per year, are associated with residential radon exposure [59]. While indoor residential radon exposure accounts for the majority of radon‐attributable lung cancer cases, occupational exposure, particularly among underground miners exposed to high radon gas concentrations, is also a considerable factor. A pooled analysis of cohort studies of lung cancer mortality among 57 873 male uranium miners from five countries reported an increasing relative rate of lung cancer with cumulative exposure to radon gas and its decay products (Estimated Relative Rate/100 Working Level Months of 1.33; 95% CI, 0.89–1.88) [60].

## 4 Recommendations for individuals

### 4.1 Scientific justification for update of the recommendations on ultraviolet radiation exposure in ECAC5

The latest evidence on UVR and cancer was reviewed to update the ECAC4 recommendation: *Avoid too much sun, especially for children. Use sun protection. Do not use sunbeds* [1].

#### 4.1.1 Evidence on the association between exposure to ultraviolet radiation and cancer

The IARC Monographs (Volume 100, Part D) classified UVR as ‘carcinogenic to humans’ (Group 1) and elevated the classification of UV‐emitting tanning devices usage to Group 1 [61]. As both solar and artificial forms of UVR are classified as Group 1 carcinogens, they demonstrate sufficient evidence to remain in ECAC5. The carcinogenicity and mechanisms of solar and artificial UVR exposure are detailed in Greinert et al. [10].

Evidence published since the publication of ECAC4 in 2014 has further elaborated the association between UVR and skin cancer. For NMSC, the risk of SCC tends to increase with cumulative lifetime UVR exposure and typically develops on chronically sun‐exposed skin, such as the head and neck. In contrast, BCC shows a stronger association with intense, intermittent exposures. Canadian data from 2015 indicated that 46.2% of BCC (25 870 cases) were linked to sunburn, sunbathing and indoor tanning devices, while 17.3% (3433 cases) of SCC were linked to sunburn and indoor tanning [62]. On the other hand, CM is associated with intense, intermittent sun exposure particularly for intermittently sun‐exposed skin [63]. Evidence suggests that CM located on intermittently sun‐exposed skin occurs more frequently in individuals with a high number of naevi. In a pooled analysis of 2617 cases from the UK and Australia, the likelihood of developing CM on intermittently sun‐exposed sites, such as the trunk, was higher for individuals with high naevus counts compared with individuals with few naevi (odds ratio [OR] 6.9; 95% CI, 4.5–10.6). Whereas CM on chronically sun‐exposed skin is more strongly associated with cumulative UVR exposure. Findings from the Norwegian Women and Cancer study prospective cohort study reported that outdoor work was associated with increased CM risk on the head and neck (relative risk [RR] 2.07; 95% CI, 1.06–4.04) [64, 65].

Occupational solar UVR exposure in outdoor workers remains an important factor for skin cancer. Outdoor workers face protracted exposure leading to heightened risk of NMSC. Results from a case–control study in eight European countries published in 2016 found an increased risk of SCC (OR 2.77; 95% CI, 1.97–3.88) and BCC (OR 1.83; 95% CI, 1.80–2.96) for outdoor workers in the farming and construction sectors, compared to indoor workers [66]. Data from the French Agriculture and Cancer cohort study published in 2021 found that CM occurred at a higher rate among female agricultural workers compared to the general population (standardised incidence ratio [SIR] 1.21; 95% CI, 1.02–1.42) [67].

The increased risk of CM from UVR via the use of indoor tanning devices has been reaffirmed by recent evidence. A meta‐analysis of 36 observational studies containing 14 583 CM cases showed a strong association between indoor tanning and CM risk (RR 1.27; 95% CI, 1.16–1.39). The risk increases when the first exposure occurs at age ≤ 20 years (RR 1.47; 95% CI, 1.16–1.85) compared with never users. For NMSC, based on 10 406 cases from 18 cohort and case–control studies, the risk was also increased for users of indoor tanning devices (RR 1.40; 95% CI, 1.18–1.65).

#### 4.1.2 Presentation of the recommendation

The starting point for the ECAC5 UVR recommendation was the corresponding recommendation in ECAC4. Data obtained from the experimental study to evaluate the preliminary ECAC5 recommendations, reported in Mantzari et al. [68], and additional findings from a qualitative study on the adoption of the ECAC4 recommendations, reported in Feliu et al. [69], were taken into account when constructing the updated recommendation. Consequently, minor adaptations to enhance scientific precision and clarity for the target audience were included. The ECAC5 recommendation now reads:

Avoid too much sun exposure, especially for children. Use sun protection. Never use sunbeds.

The phrasing in ECAC4 to avoid ‘too much sun’ may be interpreted as referring simply to being outdoors without any reference to precautionary measures during hot weather conditions. The updated ECAC5 recommendation inserts the clarification of avoiding excessive sun ‘exposure’, which more precisely describes the modifiable behaviour increasing cancer risk, namely, unprotected or prolonged exposure to solar UVR [42, 70]. It aims to make clear that the key risk factor in question is the magnitude of solar UVR exposure, which is influenced by factors including skin phototype, geographical location (latitude and altitude), time of the day and season [43, 71]. The recommendation in ECAC4 stated simply ‘avoid too much sun’ without defining what would constitute ‘too much’ sun exposure for an individual. This was deliberate, given the variation in personal characteristics, such as skin phototype, location and seasonality, which makes it difficult to generate a more precise message for the general population. Building on this, and to avoid implying a safe level of solar UVR exposure while also recognising that complete avoidance is neither feasible nor desirable for health, the ECAC5 recommendation retains the wording ‘too much’. This is intended to draw attention to situations likely to cause sunburn and encourage the use of sun protection measures.

The statement on indoor tanning devices in ECAC4 ‘do not use sunbeds’ has been updated to urge individuals to ‘never use sunbeds’. This refinement is consistent with the unequivocal message from the Scientific Committee on Health, Environmental and Emerging Risks (SCHEER) report on the biological effects of UVR from the use of UV‐emitting tanning devices for cosmetic purposes, which was published in 2016. It stated that as the induction of skin cancer is stochastic, lower doses only reduce the probability of skin cancer but do not indicate a level of irradiance at which skin cancer risk may reach zero. Consequently, there is no safe limit for exposure to UVR from indoor tanning devices [20]. Therefore, the wording of the ECAC5 recommendation was deliberately modified to reflect this evidence and reduce the possibility of misinterpretation or risk minimisation by the target audience.

The remaining statements issued in ECAC4 refer to the importance of protecting children from UVR and using sun protection. Individuals are recommended to take measures to protect against sun exposure by limiting time in the sun and seeking shade; wearing protective clothing, a broad‐brimmed hat and UV‐protective sunglasses [72]. Broad‐spectrum (UVA and UVB) sunscreens with a sun protection factor (SPF) of 30+ to 50+, applied daily to sun‐exposed body parts not protected by clothing, should be used in combination with other sun protection measures as a last line of defence [73]. This advice extends to outdoor solar UVR exposure in occupational settings. For children, shade and protective clothing are key and infants should be kept out of direct sunlight. The statements on sun protection measures in ECAC5 have remained unchanged in their phrasing as they continue to be both scientifically and behaviourally relevant. Consequently, no benefit would be attained through modifying these particular statements.

##### 4.1.2.1 Aspects of equity

Matters of equity were of key importance to reflect upon during the development of all ECAC5 recommendations. In this respect, the continuation from ECAC4 of highlighting children as a key vulnerable population group is warranted due to their limited capacity to take protective action independently, plus the established evidence demonstrating that childhood is a susceptible period for UVR‐related carcinogenesis [10].

Although individuals with lighter skin phototypes are at higher risk of skin cancer than individuals with naturally darker skin or with a tendency to tan easily, the recommendation is intentionally inclusive of all phototypes to avoid misconceptions about its relevance to individuals with darker skin [70, 74, 75, 76].

Additionally, due in part to the recognition of potential economic barriers and variability of access for individuals in the EU, the recommendation does not refer explicitly to sunscreen use as a recommended action when referring to the use of sun protection measures. Nevertheless, appropriate use of sunscreen remains an important behavioural component for protection against solar UVR. Evidence suggests regular sunscreen use, when applied consistently and correctly, may be effective in reducing CM risk [50, 73, 77, 78].

##### 4.1.2.2 Suitability, actionability and acceptability of the recommendation

The recommendation promotes simple, accessible and protective behaviours that are feasible for the vast majority of people, including workers. It is consistent with existing national sun and UVR safety guidance in EU Member States, further supporting its appropriateness and acceptability in the region [5].

#### 4.1.3 Co‐benefits for prevention of noncommunicable diseases other than cancer with similar risk factors and opportunities for health promotion

UVR exposure can cause suppression of the immune function with effects on both local and systemic immunity [79]. Excessive exposure is associated with certain eye conditions such as cataracts and age‐related macular degeneration [20, 80]. On the other hand, UVB triggers vitamin D synthesis in the skin, which is the body's primary source of vitamin D and is essential for bone development and maintenance [10, 81]. For most individuals, short periods spent outdoors, depending on the season, skin type and geographical location, are sufficient to regulate the levels of vitamin D in the body. Therefore, limited sun exposure, managed carefully in line with the ECAC5 recommendation, is sufficient for maintaining the benefits from vitamin D while minimising the associated health risks [82, 83].

Shading public places reduces UVR exposure of the public (including outdoor workers) and at the same time reduces temperature in urban areas. These measures may help to mitigate health consequences related to heat stress [13, 84, 85].

### 4.2 Scientific justification for update of the recommendation on radon exposure in ECAC5

The latest evidence on indoor radon exposure and cancer was reviewed to update the ECAC4 recommendation: *Find out if you are exposed to radiation from naturally high radon levels in your home. Take action to reduce high radon levels* [1].

#### 4.2.1 Evidence on the association between exposure to radon and cancer

Radon‐222 and decay products were classified as ‘carcinogenic to humans’ (Group 1) by the IARC Monographs (Volume 100, Part D) [61]. This classification provides sufficient confidence in the evidence to support the continued inclusion of this recommendation in ECAC5. The carcinogenicity and mechanisms of radon and its decay products are explained in McColl et al., which outlines the scientific justification for the ECAC4 recommendation on indoor radon gas [25].

A 2020 meta‐analysis of 28 studies, including 13 748 lung cancer cases and 23 112 controls, confirmed earlier findings on the dose–response relationship between residential radon exposure and lung cancer risk. For every 100 Bq·m^−3^ increase in residential radon concentration, the overall risk of lung cancer increases by 11% (excess odds ratio [EOR] 0.11; 95% CI, 0.05–0.17). The association was greater for certain histological subtypes, with a 19% increase in risk for small cell lung cancer (EOR 0.19; 95% CI, 0.07–0.32), and 13% for adenocarcinoma (EOR 0.13; 95% CI, 0.01–0.25) [86]. These findings are consistent with the previously published, and well‐established, 2005 pooled analysis of individual data from 13 case–control studies of residential radon and lung cancer in nine European countries, which estimated a 16% (95% CI, 5–31%) increase in lung cancer risk per 100 Bq·m^−3^ of residential radon gas exposure [87].

A systematic review and meta‐analysis of pooled collaborative studies published in 2021 further demonstrated the dose–response relationship between residential radon and lung cancer with stratification by smoking status. An increase per 100 Bq·m^−3^ in radon concentration heightened the risk of lung cancer by 15% among never‐smokers (adjusted Excess Relative Risk [aERR] 0.15; 95% CI, 0.06–0.25) and 9% among people who have ever smoked (aERR 0.09; 95% CI, 0.03–0.16) [88]. As the baseline lung cancer risk is higher for people who have ever smoked compared to never smokers, the absolute increase is higher for ever smokers.

Research investigating possible associations between radon exposure and cancers other than lung cancer, such as leukaemia, gastric and skin cancers, remain inconclusive at present. Evidence from a German uranium miners cohort shows very limited increased risk for cancers other than lung cancer, even at very high exposure levels. This suggests that the nonlung cancer risk at residential radon exposure levels is very small [30].

Most national and international health agencies, including the WHO, currently adopt the linear‐no‐threshold (LNT) model to assess the risk of radon‐induced lung cancer. The LNT model posits that any incremental increase in radon exposure proportionally raises lung cancer risk, with no safe lower threshold below which there is no risk. The LNT approach serves as a precautionary basis for radiation protection standards worldwide. A review of existing scientific literature by the United Nations Scientific Committee on the Effects of Atomic Radiation highlighted complications in reconstructing exposure and dose for epidemiological assessments, reinforcing how dose uncertainty and retrospective reconstruction can artificially narrow or widen confidence intervals and obscure the true relationship between dose and response [89]. Despite these debates, the balance of evidence and policy remains strongly in favour of the LNT model, given the imperative of public health protection [90].

#### 4.2.2 Presentation of the recommendation

The ECAC5 recommendation on indoor radon gas builds upon the ECAC4 recommendation taking into account findings from Mantzari et al. [68] and Feliu et al. [69]. It focuses specifically on indoor residential exposure to radon gas, while occupational exposure is addressed separately in the corresponding ECAC5 recommendation described by Jochems et al. [9]. The approved ECAC5 recommendation on indoor radon reads:

Inform yourself about radon gas levels in your area by checking a local radon map. Seek professional help to measure levels in your home and, if necessary, reduce them.

The recommendation is structured according to the practical steps to be taken to understand and make an informed decision about potentially reducing radon exposure. It encourages individuals to adopt proactive, information‐seeking behaviour, while emphasising that remediation measures themselves are conditional. This can help to avoid raising anxiety and alarm among individuals whose dwellings have low radon concentration.

Each message reflects the sequence of events in the order they are intended to occur. The first step encourages individuals to consult local radon maps to become informed about the radon concentration in their area. The inclusion of ‘radon maps’ in the ECAC5 recommendation is critical as it offers individuals an entry point to learn more about their potential residential radon exposure, in line with the requirements of the Basic Safety Standards Directive [39, 40].

While local radon maps provide valuable information regarding geographic variations in exposure, they lack sufficient resolution to determine radon concentrations at the level of individual dwellings. Therefore, some national authorities have added supplementary queries, such as foundation type or year of construction, alongside their national radon map, for example in Switzerland [91]. Accurate assessment of residential radon concentrations requires *in situ* measurements to be taken over a period of time. The ECAC5 recommendation acknowledges this requirement emphasising the importance of engaging trained professionals to ensure proper installation of test kits, reliable measurement procedures and accurate interpretation of results. Additionally, informing people to ‘seek professional help’ can help to facilitate appropriate remediation methods where necessary and to avoid the implementation of ineffective and potentially counterproductive measures [92, 93].

The final step of the recommendation is to take action to reduce indoor radon concentration only ‘if necessary’. This aligns with national radon action plans, which typically recommend remediation only when indoor radon concentration exceed national reference levels. In this way, the recommendation provides clear guidance without causing undue concern among individuals whose residential exposure does not warrant action [94].

##### 4.2.2.1 Aspects of equity

The development of the ECAC5 recommendation considered the potential equity implications across the EU. Maintaining radon as a recommendation in ECAC may help raise further awareness of radon exposure and its associated cancer risk, which could encourage greater uptake of testing and remediation, where needed. This has the potential to positively influence equity, particularly for individuals living in radon‐affected areas who may otherwise be unaware of their exposure. However, challenges remain, especially for individuals living in rented accommodation or property owners, who may face barriers to testing or remediation due to limited financial means and may not prioritise undertaking necessary remediation measures. In this context, the corresponding ECAC5 recommendation for policymakers becomes especially important to implement.

##### 4.2.2.2 Suitability, actionability and acceptability of the recommendation for the individual

The ECAC5 recommendation on indoor radon gas has been evaluated in terms of its suitability to the general population in EU Member States, its capacity to support individual action, and the acceptability of its messages. Given that radon exposure occurs at varying concentrations across all Member States, the recommendation is broadly relevant for the general population and appropriately raises awareness of the association between radon gas and lung cancer.

Evidence suggests that including a ‘call to action’ can increase emotional engagement and may improve uptake of public health guidance [95]. Consequently, the phrasing of the recommendation deliberately emphasises the sequential, actionable steps to be taken by individuals. This stepwise framing enhances the actionability of the recommendation and may in turn help to improve its acceptability by empowering individuals with concrete steps to follow.

A Canadian study in 2021 reported that only 20% participants obtained a radon test kit following a single encounter with public health information designed to raise awareness of radon gas, with 65% of participants requiring multiple follow‐up interactions before obtaining a kit. Delays in obtaining a kit and anxieties raised upon presentation of the information on radon gas varied by age, sex and occupation [96]. Variation across demographic groups suggests that different approaches are required to tailor messaging, frequency and format to the needs of specific groups. Therefore, authorities should consider to develop targeted information campaigns to more effectively address their populations on this topic.

#### 4.2.3 Co‐benefits for prevention of noncommunicable diseases other than cancer with similar risk factors and opportunities for health promotion

Reducing exposure to radon has cobenefits for health beyond cancer prevention. Efforts to lower indoor radon gas concentration, such as addressing structural gaps at the basement level and better ventilation, will also improve indoor air quality and may help alleviate respiratory conditions such as asthma [97]. As noted in McColl et al., the majority of radon‐induced cancers occur among people who smoke due to the combined effect of smoking and radon exposure and reflecting their substantially higher baseline lung cancer risk [25, 87]. Recent estimates from Germany have underscored this synergy by reporting that approximately 80% of radon‐attributable lung cancers occur among current or former smokers [56]. Therefore, the ECAC5 recommendation can also be used to reinforce broader tobacco control objectives, complementing actions on smoking cessation.

## 5 Recommendations for policy‐makers

Effective cancer prevention requires a dual‐approach combining individual‐level behavioural strategies with structural interventions that enable environments in which individuals can adopt cancer prevention guidance [98]. Policymakers are key to this process as they have the authority to regulate and promote public programmes conducive to cancer prevention [99].

In recognition of this, ECAC5 introduces a complementary set of 14 policy recommendations, which reinforce those for individuals. The recommendations reflect authoritative international policies selected according to the IARC methodology [3] and that are also supported to some extent by Europe's Beating Cancer Plan (EBCP) [100] and the WHO's NCD Best Buys [101].

### 5.1 Presentation of the recommendation for policymakers: Ultraviolet radiation exposure

Table 1 shows the adopted ECAC5 policy recommendation on UVR exposure, which includes a series of priority actions for policymakers.

**Table 1:** European Code Against Cancer, 5th edition: recommendations for policymakers on sun and ultraviolet radiation exposure.

Sun and ultraviolet (UV) radiation exposure
• Harmonise and enforce policies and recommendations on protection from exposure to UV radiation across the EU
• Continue to support measures to reduce exposure to UV radiation in the public and especially in children, including from sunbeds and excess solar UV radiation
• Provide collective protection from sun exposure, such as shading infrastructures and greening, at the local level
• In the workplace, provide organisational measures, shading and access to UV‐safe clothing or other collective and individual protective equipment to reduce exposure of workers to solar and artificial UV radiation
• Complementing the above‐mentioned policy measures, invest in and promote regular public health campaigns to raise awareness and knowledge of exposure to UV radiation and cancer risk, and monitor their effectiveness in changing behaviour and reducing exposure

© 2026 International Agency for Research on Cancer / WHO. Used with permission.

References: • Directive 2006/25/EC of 5 April 2006 on the minimum health and safety requirements regarding the exposure of workers to risks arising from physical agents (artificial optical radiation). *OJEU*. 2006;**L114**:38–59. Available from: https://eur-lex.europa.eu/legal-content/EN/TXT/?uri=CELEX:32006L0025 [102]. • Directive 89/391/EEC of 12 June 1989 on the introduction of measures to encourage improvements in the safety and health of workers at work (OSH “Framework Directive”). *OJEU*. 1989;**L183**:1–8. Available from: https://eur‐lex.europa.eu/legal‐content/EN/TXT/?uri=CELEX:01989L0391‐20081211 [103]. • SCHEER (Scientific Committee on Health, Environmental and Emerging Risks), Opinion on Biological effects of ultraviolet radiation relevant to health with particular reference to sunbeds for cosmetic purposes, 2016. Available from: https://ec.europa.eu/health/scientific_committees/scheer/docs/scheer_o_003.pdf [20].

The recommendation calls upon policymakers to adopt a harmonised approach towards protecting the population from UVR exposure across the EU. While many Member States have issued national guidelines, such as the German Guideline on Skin Cancer Prevention [104], there is currently no EU‐wide strategy to prevent harmful UVR exposure, resulting in a patchwork of national recommendations. A coordinated EU‐level framework could facilitate alignment and enhance the effectiveness of existing prevention policies through shared experiences and best practices.

Published in 2021, EBCP highlighted the need to explore measures targeting UVR exposure, with special attention to artificial UVR [100]. Therefore, the recommendation urges policymakers to address artificial UVR exposure from indoor tanning devices. Justification stems from the SCHEER opinion (2016), which reported that there is no safe level of UVR exposure from indoor tanning devices [20]. Such devices are currently regulated under the EU Low Voltage Directive, which focuses on product safety more than public health concerns [105]. WHO has outlined various evidence‐based regulatory options for governments, including either a complete ban on the use of indoor tanning devices for cosmetic purposes or control measures that are coupled with stringent requirements for informed consent [106]. Countries such as Brazil and Australia have already implemented outright national bans on these devices for cosmetic use [107]. A 2025 report has provided an overview of regulatory measures taken across the EU [108]. In Ireland, for example, the Public Health (Sunbeds) Act 2014 introduced comprehensive restrictions on the use of indoor tanning devices, which included a prohibition on use by minors aged < 18 years, mandatory health warnings, and a ban on promotional pricing [109]. As of 2022, 16 EU Member States have prohibited use by minors aged < 18 years [21]. This highlights the variability in regulatory approaches across the EU, which may be addressed through harmonised legislation. Although the European Parliament has supported the calls for EU legislation [110], at present the European Commission has yet to bring forward a Commission Recommendation on reducing the health risks associated with the use of indoor tanning devices.

To reinforce the individual‐level recommendations, measures in the built environment to reduce solar UVR exposure are important for protecting the general public and affected occupational groups. In Finland, the Radiation and Nuclear Safety Authority promotes shade provision in nurseries, school grounds and recreational areas, noting that such measures can reduce solar UV radiation by up to half compared to direct sun exposure [111]. The ECAC5 recommendation for policymakers recommends that shade provision is introduced, particularly in spaces for children and young people, throughout the EU.

The ECAC5 recommendation specifically encourages policymakers to adopt protective measures for workers exposed to UVR of any kind. Two EU directives are relevant in this context. Firstly, Directive 2006/25/EC on artificial optical radiation sets exposure limit values to protect workers against ocular and skin damage from artificial UVR but does not extend to solar radiation [102]. Nonetheless, the Occupational Safety and Health (OSH) Framework Directive (Directive 89/391/EEC) establishes general principles for workers' risk prevention, which apply to any hazards at work including natural sources of UVR. This directive states that employers have the responsibility to assess and manage UVR‐related occupational risks in accordance with the established hierarchy of control measures [103].

Finally, the ECAC5 recommendation encourages the implementation of public health campaigns and other awareness‐raising initiatives. These should be targeted (e.g. age‐relevant), evidence‐based, and implemented on a regular basis to build and maintain public understanding of UVR‐related risks and encourage protective behaviours [112]. Particular attention should be given to children, who are especially vulnerable due to their increased susceptibility and cumulative lifetime UVR exposure. As such, interventions in educational and childcare settings that involve families are especially warranted.

#### 5.1.1 Feasibility and resources required to implement the recommendation

The actions set out in the recommendation are operationally feasible and rooted in existing policies, providing a solid foundation for implementation at national and local levels across the EU.

While the resources required will vary depending on the specific context and scale of implementation, there is evidence to support a strong return on investment for skin cancer prevention initiatives consistent with the recommendation [113]. For example, in Belgium, primary prevention initiatives have been estimated to yield savings of €3.60 for every €1.00 invested by public health authorities [114].

Policymakers may also consider going further than the actions outlined in the recommendation. Although ECAC5 does not explicitly advocate for an outright ban on the use of indoor tanning devices, economic modelling has indicated that such a measure could result in greater long‐term economic benefits. In particular, healthcare savings and productivity gains have been estimated to be approximately up to three times higher under scenarios involving a complete commercial ban, compared to policies limited to restricting use by minors [115].

Overall, while some measures in the recommendation require initial investment, their implementation is feasible and justified by the anticipated long‐term public health and economic and social benefits.

### 5.2 Presentation of the recommendations for policymakers: Radon exposure

Table 2 shows the adopted ECAC5 policy recommendation on indoor radon gas.

**Table 2:** European Code Against Cancer, 5th edition: recommendations for policymakers on indoor radon gas.

Indoor radon gas
• Enforce basic safety standards for the protection of individuals' health against radon exposure. Adapt the existing EU Directive on ionising radiation to include alpha radiation emitters such as radon as a source of ionising radiation in building materials
• Develop general awareness programmes for radon, make user‐friendly tools available that include radon prediction maps at the residential, school and workplace level, and increase population‐based radon testing
• Provide financial support for radon remediation in homes and other buildings.
• Invest in training of recognised public and private bodies for workplace and residential radiation protection

© 2026 International Agency for Research on Cancer / WHO. Used with permission.

References: • Directive 2013/59/EURATOM of 5 December 2013 laying down basic safety standards for protection against the dangers arising from exposure to ionising radiation. *OJEU*. 2014;**L13**:1–73. Available from: https://eur-lex.europa.eu/eli/dir/2013/59 [39]. • Protection against exposure due to radon indoors and gamma radiation from construction materials — Methods of prevention and mitigation, IAEA‐TECDOC‐1951. Vienna: International Atomic Energy Agency; 2021. Available from: https://www-pub.iaea.org/MTCD/publications/PDF/TE-1951web.pdf [92].

A core priority for policymakers is the enforcement of basic safety standards to reduce radon‐related cancer risk. At the EU level, the Basic Safety Standards (BSS) Directive (Council Directive 2013/59/EURATOM) sets out the essential requirements for protection against the dangers arising from exposure to ionising radiation [39]. The BBS Directive covers all major forms of ionising radiation, both natural and artificial, to protect workers, the public, and patients from exposure risks. The forms of ionising radiation addressed by this Directive include alpha and beta particles, gamma rays, X‐rays, neutron radiation and cosmic radiation. The Directive applies to radiation from radioactive materials including naturally occurring radionuclides such as radon, nuclear installations, X‐ray machines and cosmic sources. It covers exposure in occupational, medical, and public settings and addresses all types of exposure situations: planned, existing (such as radon in buildings) and emergency situations. The scope specifically includes both particle and electromagnetic forms of ionising radiation, in line with scientific recommendations from the International Commission on Radiological Protection (ICRP) [116]. The ECAC5 recommendation highlights the current gap in the Directive, whereby recognised sources of ionising radiation exposure in building materials do not comprehensively address radon and its decay products. Currently, the Directive focuses on gamma radiation emitters from building materials but does not explicitly regulate alpha‐emitting radiation, such as radon which may emanate from building materials and add to indoor radon concentrations [27]. Including alpha‐emitting ionising radiation from building materials in the Directive would help to enable clearer assessment, control and labelling of implicated materials [27, 117].

Under the BSS Directive, all Member States are required to establish National Radon Action Plans, which must detail how indoor radon concentration, notably those exceeding the reference level of 300 Bq·m^−3^, is measured, reported and addressed via access to testing and remediation. All EU Member States have now adopted national plans; however, the level of implementation varies. Introducing harmonised and quantifiable indicators for measurement, mitigation and public awareness may help to better evaluate progress and support national implementation [118].

The upfront cost of radon mitigation remains a major barrier for many individuals [119]. Consistent with the spirit of the BSS Directive, policymakers are encouraged to establish or expand financial support schemes, particularly for those individuals residing in high radon areas and for low‐income households, as part of National Radon Action Plans. Several Member States have introduced such measures. For example, in Sweden and Finland, homeowners can claim tax deductions for radon remediation. In the Czech Republic, public buildings with radon concentrations exceeding 300 Bq·m^−3^ are eligible for financial support with remediation [94].

The ECAC5 recommendation for policymakers highlights the importance of complementing ongoing indoor radon measurement campaigns with sustained public awareness initiatives in Member States. Despite the well‐documented risks of radon exposure, public awareness remains limited, particularly regarding testing and mitigation strategies [120, 121]. Evidence indicates that one‐off information campaigns often fail to achieve high uptake of testing and remediation [96]. Therefore, this underlines the need for continuous, targeted communication strategies across the EU.

#### 5.2.1 Feasibility and resources required to implement the recommendation

The actions recommended for policymakers are technically feasible, financially justifiable and consistent with EU policy and technical programmes. Policymakers should prioritise investment in targeted subsidy schemes and tailored communication strategies to support adherence to regulatory standards and deliver equitable public health benefit.

With the support of the existing EU regulatory framework, monitoring, reporting and testing of indoor radon are both practical and affordable. Radon test kits typically cost under €50, and several Member States already subsidise or provide free kits, particularly in known high radon areas [122]. Additionally, technical support for radon mapping and risk assessment is available from the JRC's European Indoor Radon Map programme.

In terms of remediation, certain interventions, such as indoor ventilation improvements, are likely to involve only minor costs. Other interventions, such as structural modifications, are likely to require considerable upfront investment but still deliver savings in the long term [123]. Nevertheless, costs associated with the remediation of existing buildings can vary across Member States and can be substantial, especially where complex structural work is required. Without financial support via subsidy programmes, upfront costs may deter individuals, housing associations and corporate entities from taking necessary action to reduce indoor radon concentration [37].

## 6 Conclusions

UVR and radon gas are well‐established environmental and occupational carcinogens that remain important contributors to the cancer burden in the EU and globally. Both are classified as Group 1 carcinogens by the IARC Monographs. In line with this and in accordance with the IARC Methodology [3], ECAC5 maintains separate recommendations addressing UVR and radon gas exposures.

The recommendations have been carefully reviewed and refined to enhance their clarity, scientific precision and behavioural relevance. The recommendation on UVR exposure has been updated to emphasise the need to limit sun exposure, especially in children, encourage sun protection and firmly advise individuals to never use indoor tanning devices. The recommendation on indoor radon gas adopts a sequential approach, guiding individuals to consult local radon maps, then seek professional measurement when pertinent and undertake remediation if necessary.

For the first time, ECAC5 introduces complementary recommendations for policymakers [4], recognising the critical role of structural and regulatory interventions. For UVR, this includes advancing harmonised EU‐level policies related to UVR exposure, more stringent regulation of indoor tanning devices, and improved protection of workers exposed to both solar and artificial forms of UVR. For indoor radon gas, the recommendation calls for strengthened implementation of National Radon Action Plans, expansion of financial support and subsidy schemes, and investments in public awareness initiatives. Taken together, the ECAC5 recommendations present a comprehensive, integrated framework for addressing these key and widespread environmental carcinogens. Their adoption and implementation can contribute towards reducing preventable cancers in the EU and support the goals of Europe's Beating Cancer Plan.

## Conflict of interest

None to declare. Where authors are identified as personnel of the International Agency for Research on Cancer/World Health Organization, the authors alone are responsible for the views expressed in this article and they do not necessarily represent the decisions, policy or views of the International Agency for Research on Cancer/World Health Organization.

## Author contributions

DR, QC and RG were responsible for writing and conceptualising the first version of the manuscript. All authors gave critical revisions on the intellectual content of the manuscript and approved the final manuscript.

## Supporting information


**Fig. S1.** Global total‐sky UV Index forecast for 12:00 UTC on 13 June 2025, produced by the Copernicus Atmosphere Monitoring Service (CAMS).
**Annex S1**. European Code Against Cancer, 5th edition. © 2026 International Agency for Research on Cancer / WHO. Used with permission.

## Data Availability

The data that support the findings of this study are available in the figures and tables of this article.
