# Supplementary material for: European Code Against Cancer, 5th edition – ultraviolet radiation, radon and cancer
Source: Mol Oncol. 2026 Jan 16;20(1):49–67. doi: 10.1002/1878-0261.70171 (PMC12809468; doi:10.1002/1878-0261.70171)
Supplement: Supplementary file 1 — Fig. S1. Global total‐sky UV Index forecast for 12:00 UTC on 13 June 2025, produced by the Copernicus Atmosphere Monitoring Service (CAMS). Annex S1. European Code Against Cancer, 5th edition. © 2026 International Agency for Research on Cancer / WHO. Used with permission. [file MOL2-20-49-s001.zip › Supporting information legends.docx]

**Supporting information**

**Annex 1** – **European Code Against Cancer, 5^th^ edition (ECAC5)**

14 ways you can help prevent cancer

**Supplementary Figure S1: Global total-sky UV Index forecast for 12:00 UTC on 13 June 2025, produced by the Copernicus Atmosphere Monitoring Service (CAMS).**

Generated from Copernicus Atmosphere Monitoring Service (CAMS). UV index forecasts [Internet]. European Centre for Medium-Range Weather Forecasts (ECMWF); 2025. Available from: <https://atmosphere.copernicus.eu/charts/packages/cams/products/uvindex-forecasts>

Reuse authorised under the Creative Commons Attribution 4.0 International (CC BY 4.0) licence.
