# Supplementary figures and images for: European Code Against Cancer, 5th edition – ultraviolet radiation, radon and cancer
Source: Mol Oncol. 2026 Jan 16;20(1):49–67. doi: 10.1002/1878-0261.70171 (PMC12809468; doi:10.1002/1878-0261.70171)

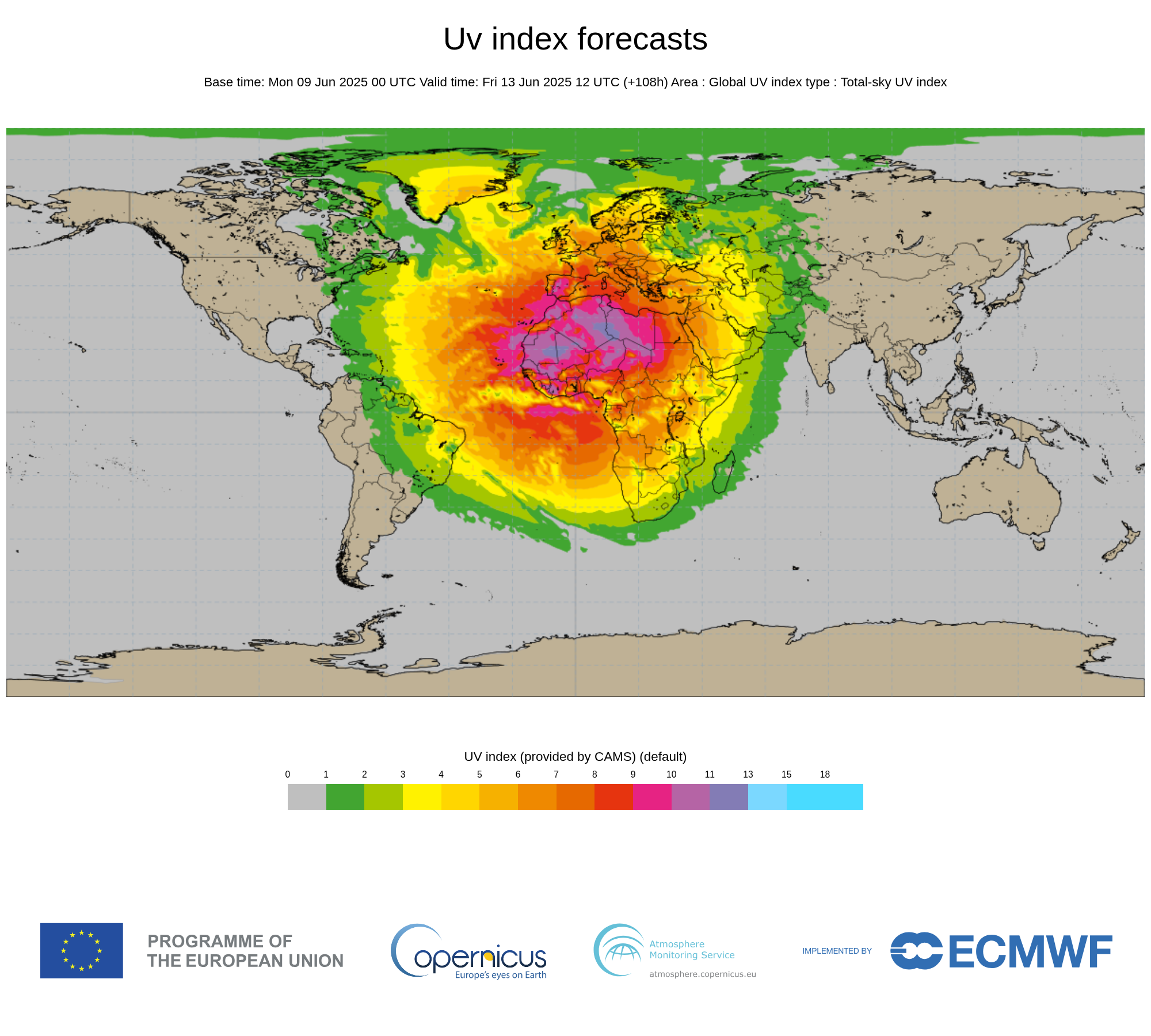

Supplement: Supplementary file 1 — Fig. S1. Global total‐sky UV Index forecast for 12:00 UTC on 13 June 2025, produced by the Copernicus Atmosphere Monitoring Service (CAMS). Annex S1. European Code Against Cancer, 5th edition. © 2026 International Agency for Research on Cancer / WHO. Used with permission. [file MOL2-20-49-s001.zip › MOL2_70171_f1_Figure_S1.tif]
